# Coming of age: - Do female harbour porpoises (*Phocoena phocoena*) from the North Sea and Baltic Sea have sufficient time to reproduce in a human influenced environment?

**DOI:** 10.1371/journal.pone.0186951

**Published:** 2017-10-20

**Authors:** Tina Kesselring, Sacha Viquerat, Ralph Brehm, Ursula Siebert

**Affiliations:** 1 Institute for Terrestrial and Aquatic Wildlife Research (ITAW), University of Veterinary Medicine Hannover Foundation, Büsum, Germany; 2 Institute for Anatomy, University of Veterinary Medicine Hannover Foundation, Hannover, Germany; Institute of Deep-sea Science and Engineering, Chinese Academy of Sciences, CHINA

## Abstract

The harbour porpoise is the only cetacean species native to German waters. Since human pressures are suggested to shorten their reproductive lifespan, basic knowledge on reproduction is strongly required. One parameter is the onset of sexual maturity in female harbour porpoises. Therefore, we investigated the first signs of sexual maturity for a period of almost two decades (1990–2016). Ovaries from 111 female harbour porpoises from the German North Sea and Baltic Sea were examined for the presence and morphological structure of follicles, *corpora lutea* and *corpora albicantia*. Based on the ovarian characteristics we performed the first model-based estimation of age at sexual maturity for harbour porpoises from German waters. Additionally, we produced a demographical age structure based on all female strandings and bycatches from German coasts. Our results showed that *corpora lutea* and *corpora albicantia* as signs of former ovulation could be found in individuals at an age of 4.95 (± 0.6) years. No significant differences between specimens from the North Sea and Baltic Sea were detected. However, the average age at death differed significantly with 5.70 (± 0.27) years for North Sea animals and 3.67 (± 0.30) years for those in the Baltic Sea. Growing evidence exists that the shortened lifespan of Baltic Sea harbour porpoises is linked to an anthropogenically influenced environment with rising bycatch mortalities due to local gillnet fisheries. Thus, our findings support the idea of local management plans based on a model-based detection of age at sexual maturity and considering the anthropogenic impacts on the population for effective protection of harbour porpoises and the North Sea and Baltic Sea.

## Introduction

The harbour porpoise (*Phocoena phocoena*) is one of the smallest cetacean species and common in shallow coastal waters of the Northern hemisphere [1–3]. The mating season is considered to occur between June and September, the birth period from June to August [4–6]. Since harbour porpoises give birth only once a year, they are considered as a slowly reproducing species [7] and therefore depend on successful mating seasons. The age of sexual maturity is an important parameter for estimating the reproductive cycle of a species and for assessing the reproductive potential in a population. In general, sexual maturity is reached when an individual is capable of taking part in reproduction. For female mammals, this coincides with the first ovulation event [8]. Female harbour porpoises from areas outside the German North Sea and Baltic Sea attain sexual maturity at the age of 3–6 years, depending on the methodology used and which sub-population was assessed [5, 9–14]. In harbour porpoises from Danish waters including the North Sea and Baltic Sea (1985–1991), sexual maturity was determined as starting at the age of 3.63 years [5]. Specimens from West Greenland (1988–1995) showed an average age at sexual maturity of 2.45 years [13], while porpoises from the Bay of Fundy, Canada (1985–1988) reach sexual maturity between the age of 3.15 and 3.44 years [11]. In Scottish waters (1992–2005), harbour porpoises were found to be sexually mature at 4.35 years [14]. In Dutch waters, early investigations (1955–1978) revealed that females are considered to be mature at an age of 6 years [9]. Examining *follicular* activity and the presence and consistency of structures found on the ovaries, such as *corpora lutea* and *corpora albicantia*, is the most recommended way to monitor the individual status of reproduction and sexual maturity [8, 15]. Mammalian ovaries contain large numbers of follicles, which undergo a maturing process and release the oocyte [16, 17]. If not followed by ovulation, a follicle can regress and form a *corpus atreticum*. For other odontocetes, like *Globalicephalus macrorhynchus*, *Physeter macrocephalus* and *Stenella attenuata*, it is known that *corpora atretica* occur regularly at every stage of the oestrus cycle [8]. After ovulation has occurred, a *corpus luteum* is formed from residual cells of the *Graafian follicle* and functions as an endocrinal gland for the production of oestrogen and progesterone [18]. The *corpus luteum* is a distinct feature on the surface of the ovary that signals an ovulation event and thus sexual maturity [8]. After full efflorescence, a *corpus luteum* rearranges into a smaller structure on the ovarian surface and persists as a *corpus albicans*. Finally, after regressing almost completely, a small flat scar on the surface of the ovary may remain discernible for an unknown time [15, 18–21]. For other odontocetes, the attainment of the age at sexual maturity ranges according to their longevity between 2–3 years (franciscana dolphin), 7 years (bottlenose dolphin), 6–9 years (hectors dolphin) and 7–12 years (short-finned pilot whale) [22–25]. Although the harbour porpoise is the most abundant cetacean found along the beaches of the German North Sea and Baltic Sea [2], only a few studies have analysed the reproduction of females so far [2, 5, 26] and none of these undertook long-term data collections, but only carried out investigations of 3–5 year intervals [11, 13, 26]. In other species, changes in the onset of sexual maturity have been identified as possible results of shifts caused by natural or anthropogenic effects [27–30]. One major anthropogenic threat is the increasing bycatch rate in the Baltic Sea, which is suspected of reducing the living stocks of the harbour porpoise in this area [31–33]. Furthermore, toxic influx into the oceans through human activities are suspected drivers of reproductive failure [34–38] and have the potential to reduce the time span of reproduction in a species. Another aspect that is considered to be related to fetal loss and reduced fecundity (at least in other areas) is the incidence of infectious diseases such as infections with *Brucella ceti* [39]. This present study provides the first model-based estimationof age of sexual maturity of female harbour porpoises in the German North Sea and Baltic Sea considering mortalities attributable to anthropogenic causes to estimate the total number of females contributing to the reproductively active population.

## Materials and methods

### Sample collection

For determining the demographical age of subpopulations a dataset of 526 female harbour porpoises was utilised. Specimens were collected within the German stranding network, which conducts work (collect and hold carcasses and samples from European protected species) on German strandings following appropriate licenses from the relevant authorities (Ministry of Energy, Agriculture, the Environment, Nature and Digitalization, Ministry of Agriculture, Environment and Rural areas). Most of the animals were found stranded along the coast of Schleswig Holstein of the North Sea and western Baltic Sea between 1990 and 2016, only a small number of animals have been identified as bycatch (n = 159 between 1990 and 2014). All specimens were sampled during necropsies at the Institute for Terrestrial and Aquatic Wildlife Research (ITAW) in Büsum, Germany. Specimens were either dissected upon arrival or stored at -20°C prior to necropsy. All necropsies followed standardised protocols for harbour porpoises [40].

### Age assessment

**Fig 1:**
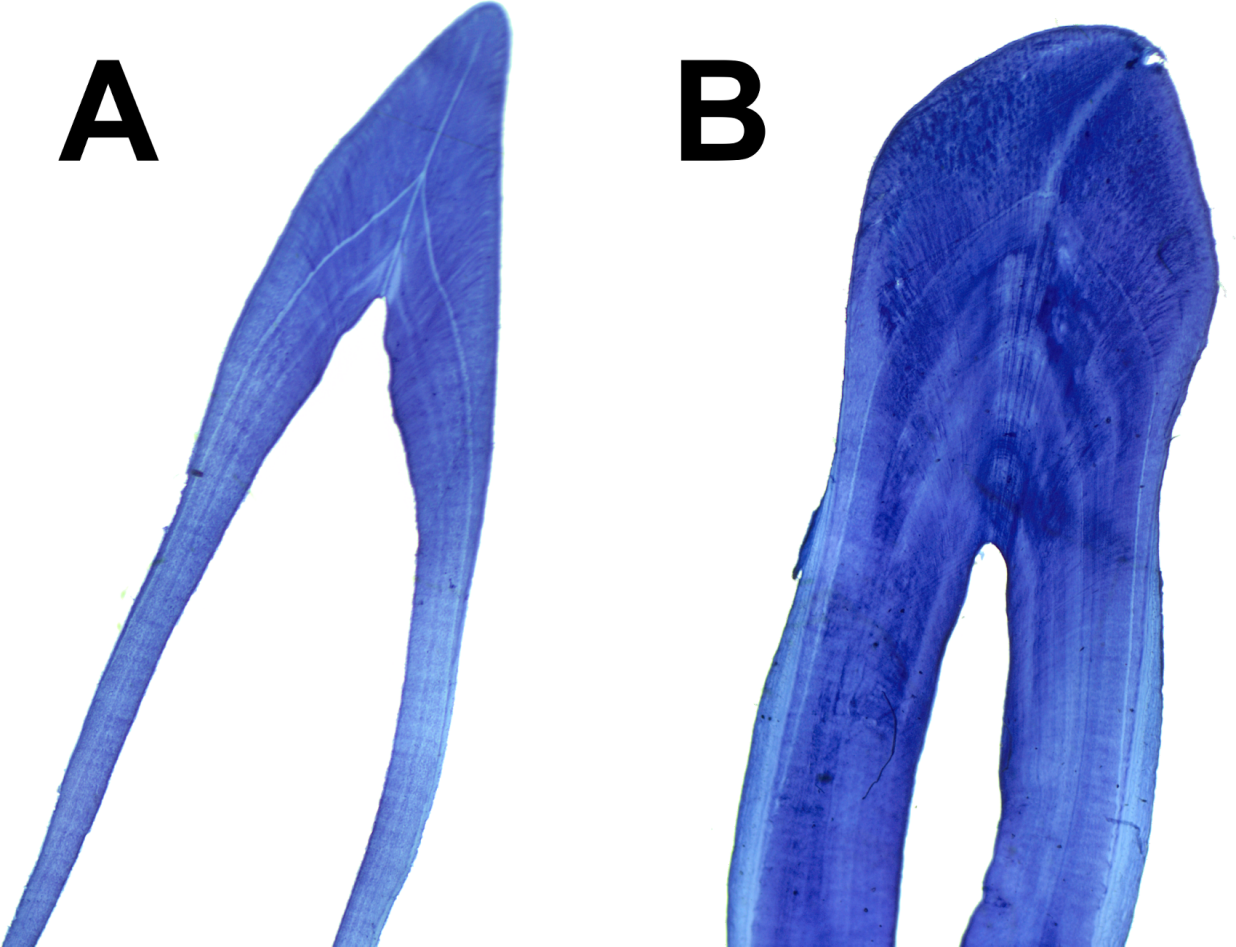
Mid-longitudinal section of a tooth from a neonate (A) and an adult (B) harbour porpoise (*Phocoena phocoena*).

The extraction, preparation, processing and age assessment followed a standard protocol [41]. At least two different readers made three independent assessments of 15 different tooth sections per individual, resulting in over 30 independent age determinations per individual (Growth Layer Groups, hereafter referred to as GLG). All readings were averaged and approximated to the nearest whole year (Fig 1).

### Ovarian examination

The reproductive tract of 111 animals was extracted, weighed and measured. A 4% phosphate-buffered formalin solution was used for fixating the samples. The ovaries were sliced into 1mm thick sections and all *corpora* and ovarian scars were counted and measured under a microscope (magnification: 4x) to the nearest 0.01mm using digital callipers. The formalin–fixed ovaries were transferred to 70% ethanol, dissected and embedded in paraffin wax using standard techniques. Sections of 5 μm thickness were stained with hematoxylin and eosin (H&E) and assessed under a light microscope. A Masson´s trichrome staining was performed for identifying connective tissue in potential *corpora albicantia*. A specimen was assumed to be sexually mature when at least one corpus could be detected on the ovaries. All examinations adhered to the procedures and terminology as recommended by the International Whaling Commission [8].

### Data analysis

In a first step, the information on the number of *corpora* per individual was translated into a binomial response value (*b_corpora*), indicating the presence or absence of *corpora* in each specimen. This was irrespective of the actual number of *corpora*, enabling us to identify the age at which we expected more than 50% of the animals to exhibit *corpora* within the ovaries (and thus, according to the above definition, to have entered the reproductive cycle).

Assuming a functional relationship between the specimen’s age and the binomial response variable *b_corpora*, we utilised a logistic regression model (formula 1) using a logit link (formula 2) testing for an effect of the North Sea and Baltic Sea specimen on the intercept in R 3.2.2 [42]:

$$L(\mu |Y)=\prod_{i=1}^{n}(1_{y_{i}=1}(\mu_{i})+1_{y_{i}=0}(1-\mu_{i}))$$ (formula 1)

where L denotes the likelihood that the predicted probabilities of the link function μ_i_ results in a success and 1_yi_ denotes the indicator function taking the value 1 if y_i_ occurs and 0 otherwise. In order to incorporate age as a covariate, we used the logit link function (formula 2):

$$\mu =g(x)=\frac{M}{1+e^{-k(x-x_{0})}}$$ (formula 2)

where M denotes the maximum of the sigmoid, k the steepness of the sigmoid, x_0_ the x-value of the sigmoid midpoint and x the observed sample values.

In a second step, we used all available data on female specimens collected between 1990 and 2016 that had undergone GLG age estimation in order to assess a demographical structure of the harbour porpoise within the study area. After grouping into age classes, we calculated the estimated proportion of animals that is part of the reproductive population at any given time, applying the result of the binomial regression model in the previous step as a threshold value. Using this model approach, covariates such as tooth age and area can be included, which would not be as trivial in an ANOVA environment e.g. due to the error distribution of the binomial response variable.

## Results

**Fig 2:**
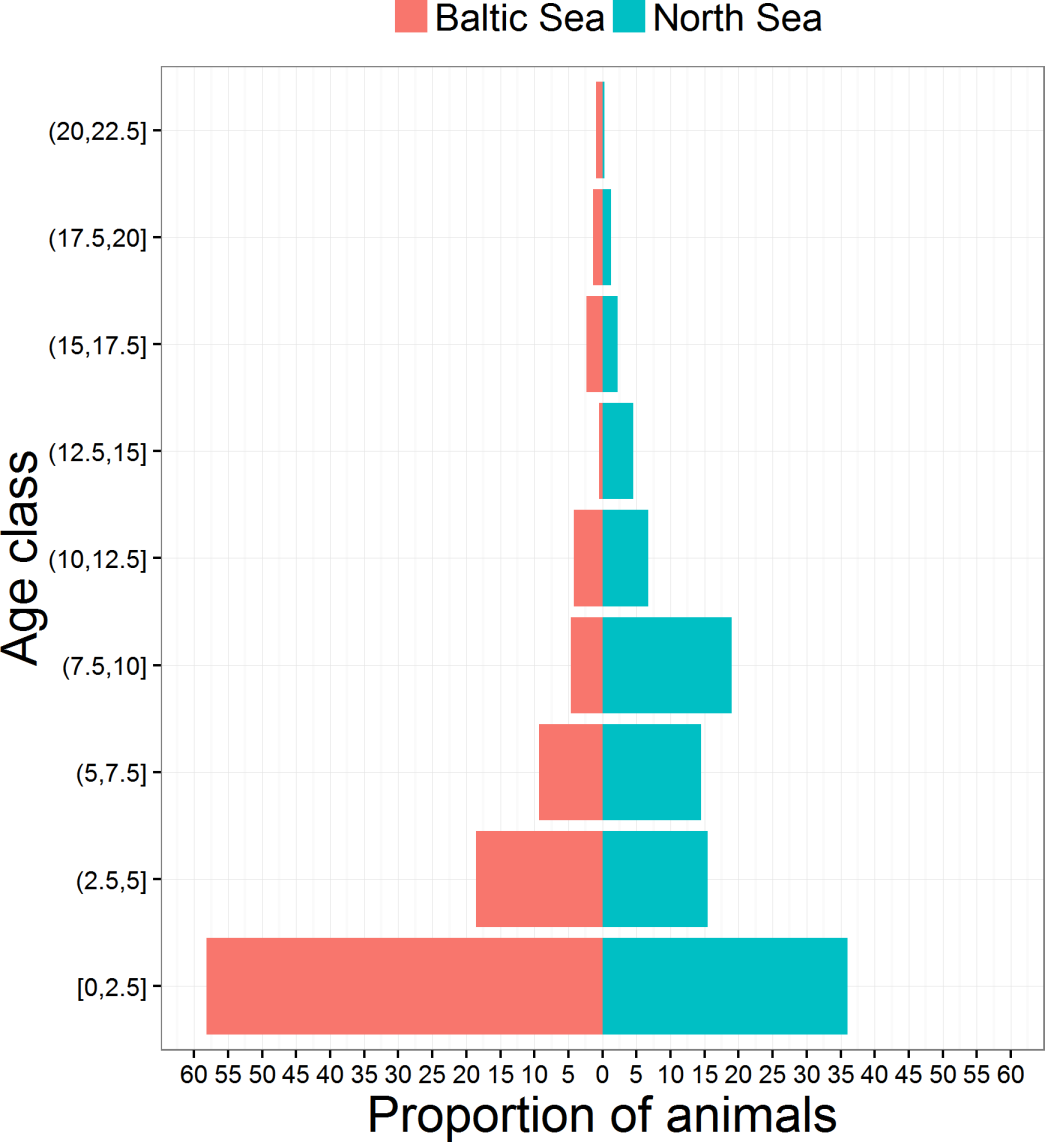
Population structure of female harbour porpoises of the North Sea (green bars) and Baltic Sea (red bars). Proportion of animals indicates the proportion of animals relative to the total number of animals within a given age bracket. Round brackets indicate exclusive values, square brackets indicate inclusive values.

We evaluated a demographic age structure based on stranding records of 526 female harbour porpoises that had had their age assessed by means of GLG (of which 311 had been found along the German North Sea shore and 215 on the German Baltic Sea shore between 1990 and 2016). The age structure ranged between 0 and 22 years, with an estimated mean age of 4.87 ± 0.20 years (Table 1). The distribution of age classes is given in Table 1 and in more detail in Fig 2. The average age at death of German North Sea specimens was 5.70 (± 0.27) years for North Sea animals and 3.67 (± 0.30) years for specimens from the German Baltic Sea, respectively.

**Table 1:** Quartile age distribution of harbour porpoises from the German North Sea and Baltic Sea between 1996 and 2017.

Area	No.	25%	50%	75%	90%	Max.	Average (± SE)
North Sea	311	1.25	5.00	9.00	12.00	22.00	5.70 (± 0.27)
Baltic Sea	215	1.00	1.6	5.0	9.6	22.00	3.67 (± 0.30)
Total	526	1.00	3.78	8.00	11.00	22.00	4.87 (± 0.20)

No. indicates number of available samples; percentage indicates the respective quartile applied to the data; max. indicates maximum observed age; average indicates respective mean age including Standard Errors (SE); all values are given in years.

**Fig 3:**
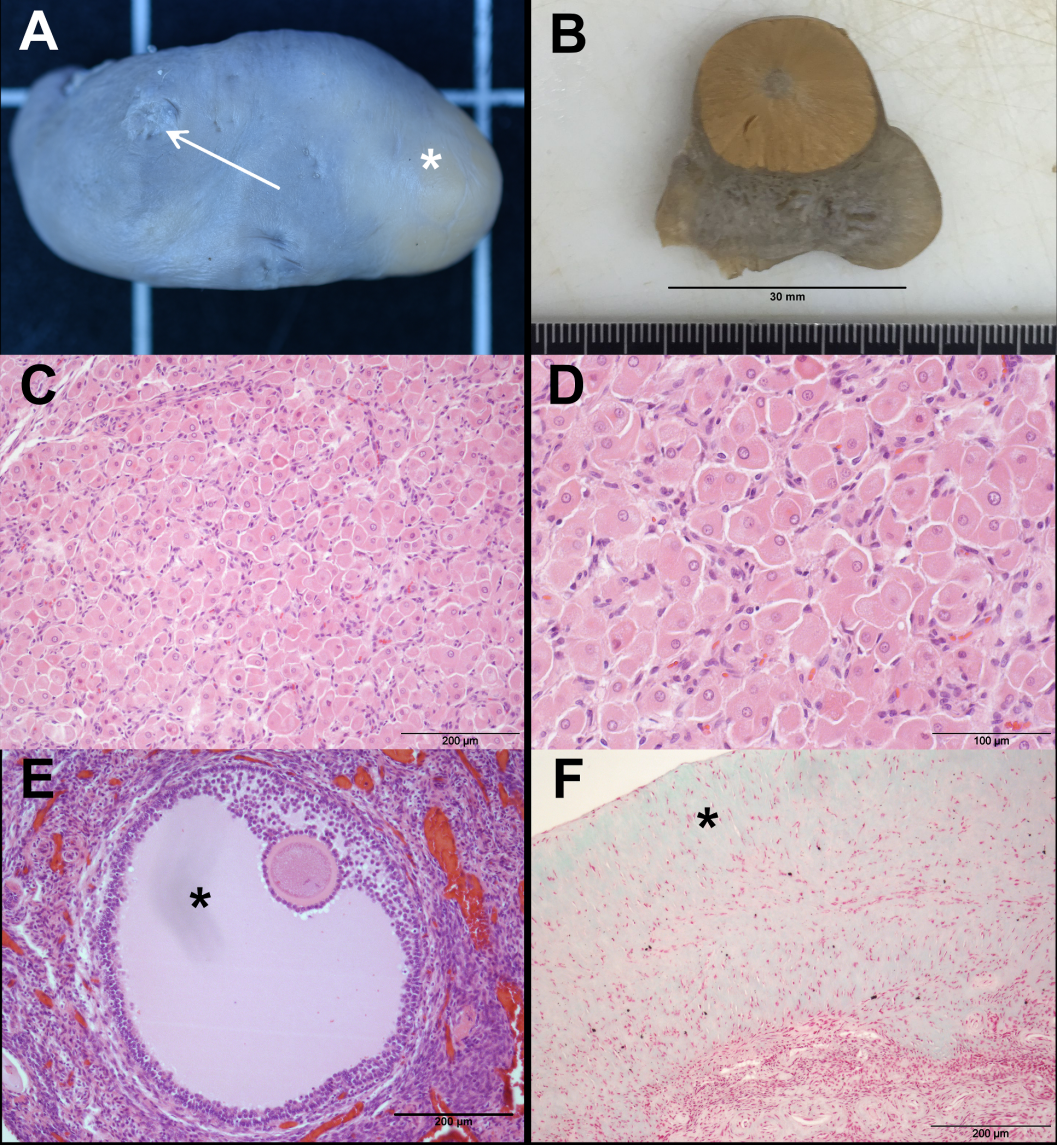
Ovary of an adult female harbour porpoise showing signs of former ovulation and tertiary follicles. (A): *Corpus luteum* (asterisk) and *corpus albicans* (arrow). (B): *Corpus luteum* as a protuberance on the ovarian surface (C), (D): Histological section through a *Corpus luteum* showing luteal cells. Staining: H&E. (E): Histological section showing the *Cumulus oophorus* with the oocyte surrounded by the *Zona pelucida* and *Corona radiata*. Asterisk: *Antrum folliculare*. Staining: H&E. (F): Histological section through a *Corpus albicans* showing an increased amount of connective tissue (asterisk). Staining: Masson´s Trichrome.

Ovaries from 111 specimens were analysed in this study (Fig 3). Of these, 69 were found along the North Sea shore (41 of which displayed at least one *corpus luteum* or *albicans*) and 42 were found along the Baltic Sea shore, of which 18 specimens providing at least one *corpus luteum* or *albicans* (Table 2). Age determination was available for all 111 animals through GLG.

**Table 2:** Overview of analysed specimens.

Area	Corpora	No.
**NS**	Absent	28
	Present	41
	Total	69
**BS**	Absent	24
	Present	18
	Total	42

Area: Origin of the specimen (NS: German North Sea, BS: German Baltic Sea); Corpora: Binary value indicating the presence or absence of corpora on the ovaries; No.: Total number of specimens assessed.

**Fig 4:**
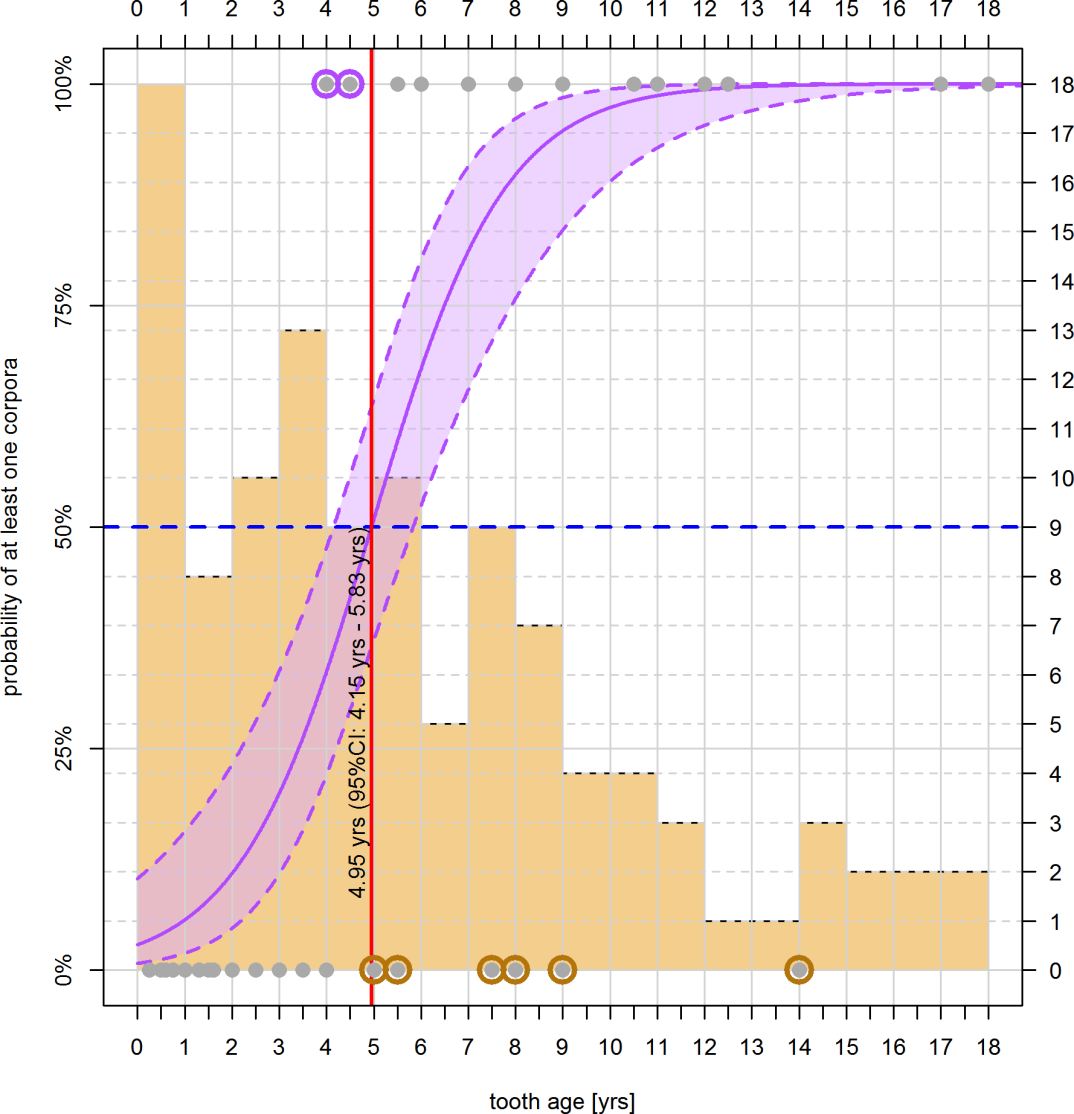
Binomial regression model showing the functional relationship between the presence of corpora (purple polygon) in female harbour porpoises from the German North Sea and Baltic Sea in relation to their age as determined by GLG. The filled area indicates the 95% confidence interval of predicted values. The red line within that area indicates the predicted average probability of finding corpora within specimens at the given age; the blue dashed line marks a probability of 50%, with the red solid line indicating the corresponding age based on the intersection of the blue dashed line with the x-axis; samples are indicated as dark grey dots; a purple circle around data points indicates animals below the age threshold showing at least one corpus, an orange circle indicates animals above the threshold showing no corpora; background histogram (orange) shows number of animals (secondary y-axis) across tooth age classes (at 1-year intervals).

Using model c2 (Table 3), we predicted the threshold age at which more than 50% of the specimensdisplayed one corpusor more as a sign of formerovulation. The threshold was determined at 4.95 years or higher (95% CI: 4.2–4.8 years, Fig 4).

**Table 3:** Model diagnostics for the binomial regression model of the probability of detecting corpora (models c1 and c2).

Response	Model name	AIC	Parameter	Sig.
**Presence of corpora**	c0	155.44	intercept	0.51
	c1	83.304	intercept	***
			tooth age	***
			area	0.174
	**c2**	**83.256**	**intercept**	***
			**tooth age**	***

Response: the response variable for the model; model name: The identifier for the model as used in the text (model name in bold indicates the chosen model); AIC: Akaike Information Criterion [42] to assess model fit; covariate: Individual covariates tested in the model (covariates in bold show significance at α = 0.05; Parameter: Name of the model parameter (c0 is the null model, i.e. assuming a uniform distribution of data without covariates); Sig.: Significance level (*** p-value ≤ 0.001).

Assuming that the animals enter their reproductive cycle at 4.95 years of age (as indicated by the previous analysis step), we estimated a total of 45.34% of female harbour porpoises in the North Sea and 72.56% in the Baltic Sea to participate in reproduction (Table 4).

**Table 4:** Percentile of all animals found between 1990 and 2017 that were older than the estimated threshold age of reaching sexual maturity of the population.

**Area**	**No.**	**Above threshold**	**P**
**North Sea**	311	141	45.34%
**Baltic Sea**	215	156	72.56%
**Total**	**526**	**297**	**56.46%**

No.: Total number of samples available; above threshold: Number of animals that were older than the threshold identified in this study; P: Proportion of animals that are above the threshold.

## Discussion

This study presents the first model-based estimation of sexual maturity using ovarian characteristics from female harbour porpoises collected from the German North Sea and Baltic Sea. Using a model approach, we identified the threshold at which more than 50% of all specimens qualify as mature without setting an arbitrary threshold that is biased by the observer. While data from stranding networks do not necessarily reflect the status of the entire population, these estimates have to be regarded as best available estimates rather than an absolute representation of the population. Using necropsy data from an unknown proportion of animals is always connected with a degree of uncertainty. We do not assume that animals found dead on the beaches represent the average population individual, nor that these stranded animals are spatially correlated to all population members and therefore might only represent a small fraction of the actual population. The onset of sexual maturity is very likely to be quite robust throughout individuals from stranding events across 20 years (as shown in the modelling step) and we therefore assume that the result is true for at least the proportion of animals that were found along the beaches. A data set spanning two decades is more susceptible to long term effects such as pollutants and individual life history events, which we aim to address once more data on GLG age estimates are collected. However, this is the best data set currently available and we must therefore assume that our results provide information on the average harbor porpoise (be they in perfect condition or under influence of pathological changes). The age of specimen ranged from 0 to 22 years, with a mean age of sexual maturity of 4.95 years. We could not detect any significant differences between specimens found in the Baltic Sea and those from the North Sea. It appears that specimens from the German Baltic Sea shore are slightly younger when reaching sexual maturity. Considering regional differences such as the magnitude of anthropogenic impacts, environmental settings and distinct prey availability, life parameters such as threshold age of sexual maturity, birth rates and calving intervals cannot be generalised for all harbour porpoises across (sub-) populations. It has been observed in other mammalian species that a good nutritional status can relate to an earlier onset of sexual maturity due to physiological features [43, 44]. Anthropogenic factors such as underwater noise, disturbance, bycatch, increasing amounts of marine debris and chemical pollution trigger changes in marine mammal physiology and manifest in an increased stress hormone level [45–47]. It has been shown that high stress levels are directly connected to the function of the hypothalamo-pituitary axis of the reproductive system of other mammals like humans, pigs and rats [48–52] and earlier onset of sexual maturity [53–55]. The direct effects on the reproductive hormonal system evoke amodified synthesis and secretion of Gonadotropin-releasing-hormone and affected responsiveness of the gonadotrophs to Gonadotropin-releasing-hormone. Furthermore, stress hormones are suspected of affecting the feedback mechanisms of steroid hormones in the hypothalamus and the pituitary gland [55]. These stress-induced effects on the reproductive system might be an evolutional value for the adaption to unfavourable environmental conditions. On the one hand, resources can be focussed on survival or improvement of an overall health status and, on the other hand, young females that might not have the potential to survive too long can be capable of producing offspring [54]. As first evidence is given on how the hormonal systems of harbour porpoises respond to stressors and in which way they might be influenced [47], a correlation between stress hormone levels and reproductive parameters in the future is strongly required in order to see whether existing knowledge from other mammals can be aligned with that of harbour porpoises. Furthermore, high levels of stress hormones are known to suppress the immune system [55], leading to an increased risk of viral and bacteriological infections [56]. These infections often cause high levels of inflammatory proteins such as cytokines that suppress fertility [57–58].

Growing evidence is given that besides the hormonal system of reproduction, also the immue system is susceptible to endocrine disrupters [59–61]. Chemical toxicants such as pesticides and plasticisers are major anthropogenic drivers of changes in the reproductive cycle of marine mammals, in particular the onset of sexual maturity [35]. PCBs (*polychlorinated biphenyls*) accumulating in the blubber of harbour porpoises from the North Sea could act as estrogenic as well as anti—estrogenic agents since they are capable of interacting with different hormonal mechanisms [60]. As an example, pituitary cells of laboratory rats exposed to contaminants show enhanced gonadotropin responses to GnRH and estrogens [62], or they are able to bind to estrogen receptors and thus inhibit or modify the cell response [63]. As such, it cannot be ruled out that changes in the onset of sexual maturity might depend on the spatial and temporal concentration of chemical pollutants within a local food web and might include a generation bias. Subsequent studies using larger datasets with additional information on potential factors that might influence reproductive parameters such as toxicant load, endocrine disruptors or marine debris findings would support a more explicit and spatial analysis of our findings. Regarding the poor availability of estimations for the age at sexual maturity in the study region, we consider the estimated mean age of sexual maturity to be a valid baseline for future investigations. This issue could support the calculation of reproductive lifespan, pregnancy rate and calving interval as indicators for the reproductive status of a population. Although our findings are in accordance with the range of observations reported from harbour porpoises from the North Atlantic, the age at sexual maturity appears to occur slighly later in the study area than in most of the other investigated regions. If we apply our age estimate of first signs of sexual maturity to the age structure of specimens found in the same region, we conclude that about 45% of animals in the North Sea and up to 73% of animals in the Baltic Sea might belong to the reproductively active part of the populations. Population estimates from the National German Monitoring Programme for harbour porpoises reveal that the population in the German North Sea contains about 50,000 animals [64] and that in the German Baltic Sea about 3,000 animals [personal communication with Sacha Viquerat]. Based on necropsy data, the sex ratio was assumed to be roughly 46% females. We infer that at peak population size in summer the total number of females belonging to the reproductive population amounted to 10,350 females in the German North Sea and only 1,007 females in the German Baltic Sea. Although the Baltic Sea reaches a higher percentage of reproductively active individuals and therefore might be capable of a faster regenaration of the population size, this value is still very low compared to other harbour porpoise populations and might be more vulnerable to environmental factors that shorten the reproductive lifespan. The low number of the Baltic Sea (sub-) populations could be caused by a variety of anthropogenic factors, especially increasing bycatches in the Baltic Sea [33]. Of the 4006 individuals, 159 were directly handed over by fishermen and classified as bycatch (~3% of all animals). 159 of 548 dissected animals were suspected to be bycaught based on pathological findings (~30% of all animals). The German stranding data showed that most of the 159 recorded bycaught porpoises between the years 1990–2014 were reported from bottom-set gillnet fisheries or stranded with characteristic net marks [46, 65]. Bycatches might affect the (sub-) populational development since female adult harbour porpoises are often bycaught due to their distribution near the shore during the reproductive period and their distinct foraging behaviours during lactation [66, 67] as well as juvenile harbour porpoises that show unestablished foraging habits.

In this study, the significantly lower age at death of individuals from the Baltic Sea compared to North Sea specimens supports the proposal to reduce the magnitude of bycatches to meet the conservation issue for harbour porpoises [33].We also observed a decline in the number of *corpora* in animals older than 8 years in samples from the German North Sea shore (but not in specimens from the German Baltic Sea, which we attribute mainly to the overall lower age of 3.67 years in Baltic Sea specimens). This decline might show, that more older signs of ovulation on the ovaries disappear than new ones develop as a potential sign of sexual senescence. This could indicate, that animals beyond that age do not succesfully mate regularly at least once a year after the age of 8 has been reached and thus, that the lifespan of full reproductive activity amounts to only about 3 years. This is a sensitive parameter with repect to human influences that might even shorten this timeframe that is essential for maintaining a constant population size. For future studies it is paramount that more data on the individual health condition and pathological findings of the investigated animals are included in the analyses to minimise this possible bias. Despite the fact that our data were too scarce to be able to conduct an elaborate analysis of the different observations in the NorthSea and Baltic Sea, it might be crucial for future investigations to consider the age at sexual maturity and corpus counts. Furthermore, other reproductive parameters such as pregnancy rate, birth rate, ovulation rate and even pathological findings in the reproductive tracts aswell as toxicological analyses to gain a complete overview of the reproductive status of a population are essential. Comprehensive long-term data including geographical information could support the analysis on a smaller spatial scale. Such datasets have been initiated for collecting from all HELCOM and OSPAR countries. Combining demographical data from specimens and population census data to estimate the proportion of sexually active animals of a population would be a key tool for any population viability study and a decisive concept to be applied in management plans. We reiterate the need for monitoring the age of sexual maturity at least bi-annually to identify any potentially serious changes in the reproductive potential of the (sub-)populations and thereby to conduct a retrospective trend analysis of data. Reporting bi-annual estimates of sexual maturity per (sub) population would be a valuable contribution to understanding population dynamics in harbour porpoises and to providing guidelines for swift policy decisions.

## Supporting information

S1 File Findings in the ovaries of female harbour porpoises. Dataset includes the origin and individual data of the carcasses and the corresponding findings on the ovaries.
(XLSX)

S2 File Age distribution of female harbour porpoises. Dataset includes individual data of the carcasses and their corresponding tooth age.
(XLSX)
