# Correction: Coming of age: - Do female harbour porpoises (*Phocoena phocoena*) from the North Sea and Baltic Sea have sufficient time to reproduce in a human influenced environment?

**DOI:** 10.1371/journal.pone.0199633

**Published:** 2018-06-20

**Authors:** Tina Kesselring, Sacha Viquerat, Ralph Brehm, Ursula Siebert

There is an error in the last sentence of the third paragraph of the Results section. The correct sentence is: The threshold was determined at 4.95 years or higher (95% CI: 4.15–5.83 years, Fig 4).

There is also an error in Table 4. The values and percentages shown within this table incorrectly report the proportion of animals below the threshold age rather than above the threshold age. Please see the corrected Table 4 here.

**Table 4:** Percentile of all animals found between 1990 and 2017 that were older than the estimated threshold age of reaching sexual maturity of the population.

Area	No.	Above threshold	P
**North Sea**	311	170	54.66%
**Baltic Sea**	215	59	27.44%
**Total**	**526**	**229**	**43.54%**

No.: Total number of samples available; above threshold: Number of animals that were older than the threshold identified in this study; P: Proportion of animals that are above the threshold.

Due to this error, several sentences within the body of the paper should also be corrected.

The final sentence of the Results section should read: Assuming that the animals enter their reproductive cycle at 4.95 years of age (as indicated by the previous analysis step), we estimated a total of 54.66% of female harbour porpoises in the North Sea and 27.44% in the Baltic Sea to participate in reproduction (Table 4).

The tenth sentence of the second paragraph of the Discussion section should read: If we apply our age estimate of first signs of sexual maturity to the age structure of specimens found in the same region, we conclude that about 54% of animals in the North Sea and up to 27% of animals in the Baltic Sea might belong to the reproductively active part of the populations.

The thirteenth, fourteenth, and fifteenth sentences of the second paragraph of the Discussion section should be replaced by the following text: We infer that at peak population size in summer the total number of females belonging to the reproductive population amounted to 12,430 females in the German North Sea and only 372 females in the German Baltic Sea. For the Baltic Sea subpopulations this value is still very low compared to other harbour porpoise populations and might be more vulnerable to environmental factors that shorten the reproductive lifespan like increasing bycatches in the Baltic Sea [33].
